# Culturing periprosthetic tissue in BacT/Alert® Virtuo blood culture system leads to improved and faster detection of prosthetic joint infections

**DOI:** 10.1186/s12879-019-4206-x

**Published:** 2019-07-10

**Authors:** Adriana Sanabria, Merethe E. O. Røkeberg, Mona Johannessen, Johanna Ericson Sollid, Gunnar Skov Simonsen, Anne-Merethe Hanssen

**Affiliations:** 1 Research Group for Host-Microbe Interactions, Department of Medical Biology, Faculty of Health Sciences, UiT, The Arctic University of Norway, Tromsø, Norway; 2 Department of Microbiology and Infection Control, University Hospital of North Norway, Tromsø, Norway

**Keywords:** BacT/Alert® Virtuo blood culture system, Blood culture bottle, Prosthetic joint infection, Periprosthetic tissue specimens

## Abstract

**Background:**

Blood culture bottles (BCBs) provide a semiautomated method for culturing periprosthetic tissue specimens. A study evaluating BCBs for culturing clinical samples other than body fluids is needed before implementation into clinical practice. Our objective was to evaluate use of the BacT/Alert® Virtuo blood culture system for culturing periprosthetic tissue specimens.

**Methods:**

The study was performed through the analysis of spiked (*n* = 36) and clinical (*n* = 158) periprosthetic tissue samples. Clinical samples were analyzed by the BCB method and the results were compared to the conventional microbiological culture-based method for time to detection and microorganisms identified.

**Results:**

The BacT/Alert® Virtuo blood culture system detected relevant bacteria for prosthetic joint infection in both spiked and clinical samples. The BCB method was found to be as sensitive (79%) as the conventional method (76%) (*p* = 0.844) during the analyses of clinical samples. The BCB method yielded positive results much faster than the conventional method: 89% against 27% detection within 24 h, respectively. The median detection time was 11.1 h for the BCB method (12 h and 11 h for the aerobic and the anaerobic BCBs, correspondingly).

**Conclusion:**

We recommend using the BacT/Alert® Virtuo blood culture system for analyzing prosthetic joint tissue, since this detect efficiently and more rapidly a wider range of bacteria than the conventional microbiological method.

**Electronic supplementary material:**

The online version of this article (10.1186/s12879-019-4206-x) contains supplementary material, which is available to authorized users.

## Background

Prosthetic joint infection (PJI) is one of the most serious complications in joint implantation, and if untreated, it may lead to severe pain, persistent dislocation and death [1]. Approximately 2 % of all patients undergoing joint replacement worldwide are affected by this complication [2–4] and this number is expected to increase with the increasing incidence of arthroplasty surgery [3, 5–7]. The early diagnosis of PJI plays a key role in successful treatment, however, the condition is difficult to diagnose [8, 9].

The diagnosis of PJI is not standardized [9]. The scheme currently in use combines clinical findings and laboratory results [10] where the microbiological assessment of periprosthetic tissue is an important criterion for diagnosis of PJI [4, 10, 11]. Most clinical microbiology laboratory diagnostic methods for PJI are based on culturing bacteria on agar plate and in enrichment broth. These methods are labor intensive, involve subculturing and require daily inspection of enrichment broths. Furthermore, low sensitivity and lack of specificity leads to 10 to 30% false-negative results [3, 12]. These numbers are not surprising. The accurate diagnosis of PJI is challenging due to various factors that affect culture results such as: low bacterial inoculum concentration, prior antibiotic therapy, formation of biofilms, and presence of fastidious slow-growing microorganisms [4, 13, 14]. Hence, improved methods for culturing periprosthetic tissue for diagnosis of PJI are urgently needed.

Inoculation of blood culture bottles (BCBs) is often used for detection of microorganisms. Clinical microbiology laboratories use this semiautomated system with continuous monitoring for microbiological diagnosis, mainly from blood and other body fluids [15]. Previous studies have demonstrated the potential of microbiological detection using BCBs for culturing specimens related to PJI, such as synovial fluid [2, 16], sonication fluid [5, 17–19], and prosthetic joint tissue (PJT) [5, 20–22]. However, there is little data on the culture of PJT samples on BCBs and more evidence is needed for safe patient management before method implementation into the clinical setting [12].

The most common BCB systems used worldwide are BD BACTEC (Becton Dickinson Instrument Systems, Sparks, MD) and BacT/Alert® (bioMérieux, Marcy l’Etoile, France) [15, 23, 24]. The systems are similar as they detect CO_2_ production and change in pH but differ in culture media composition and additives. There are many technical factors that could affect the sensitivity of blood culture systems, such as the clinical sample type, volume of blood required for culture, and timing of blood culture media, among others. In order to ensure laboratory quality practice, it is important to verify, evaluate and optimize the use of each BCB system when users practice a subprocess that differs from the manufacturer’s guidelines, such as the implementation of BCB for clinical samples other than blood [20].

To the best of our knowledge, most studies on PJI to date have used the BD BACTEC BCB system [5, 17, 19, 22]. Here, we evaluate use of the BacT/Alert® Virtuo BCB system for culturing periprosthetic tissue specimens by the analysis of spiked samples (i.e. simulated PJT specimens with known bacteria) and clinical tissue samples. The use of BCBs emerges as an attractive tool for accurate and timely diagnosis of PJI, leading to improvement in outcome for this challenging type of infection.

## Methods

### Study design and samples

A prospective laboratory study was conducted over a 11-month period (August 2017–June 2018). Periprosthetic tissue specimens from hip, knee, elbow, ankle and shoulder, belonging to patients with suspicion of PJI were routinely submitted to the Department of Microbiology and Infection Control at the University Hospital of North Norway (UNN), Tromsø, Norway.

Samples were processed using routine standard microbiological procedures. Excess tissue samples (*n* = 158) from 62 patients were evaluated through the BacT/Alert® Virtuo BCB system. The mean number of specimens received per patient was 2.5 (mode 2, range 1–5).

The BCB method was based on, and modified from, similar methods used in previous studies [12, 20, 21] while the conventional method was an already validated in-house method.

### Ethics statement

The work was performed in compliance with the ethical guidelines established by UiT- The Arctic University of Norway. The project was evaluated by the Regional Committee for Medical and Health Research Ethics, Norway (document no. 2016/1247/REK nord), concluding that ethical approval was not required. There were no ethical issues to consider due to use of anonymous clinical samples and development of methodological procedures.

### Spiking experiments

Six bacterial species reported as common microbiological causes of PJI were used for spiking experiments. *Escherichia coli* ATCC 25922, *Staphylococcus aureus* ATCC 25923, *Enterococcus faecalis* ATCC 29212, *Staphylococcus epidermidis* ATCC 12228, *Bacteroides fragilis* ATCC 25285 and *Cutibacterium* (formerly *Propionibacterium*) *acnes* (clinical sample).

Excess material of a native femoral head and surrounding tissue from an anonymous donor was crushed, sterilized (irradiated to 25Gy) and tested for contamination by culturing on agar plates. Fresh bacterial cultures were suspended in NaCl 0.85% or in tryptic soy broth (TSB) for aerobic and anaerobic strains, respectively, to a 0.5 McFarland density. These were further diluted to bacterial suspensions of approximately 10^3^ colony forming units (CFU)/mL.

A piece of sterilized crushed native femoral head and tissue (≈1 cm^3^), i.e. a simulated PJT sample, was transferred to a 15 mL tube containing four mL of glucose broth and five ceramic beads. Five hundred microliters of the bacterial suspension (≈500 CFU) was added to the glucose broth containing the tissue. After two minutes, the mixture was homogenized using a FastPrep-24 instrument (MP Biomedicals, France) to 6.0 M/sec (meters per second) for 40 s.

Subsequently, a Bact/Alert® BCB (FA Plus for aerobic or FN Plus for anaerobic bacteria, bioMérieux, Marcy l’Etoile, France) was inoculated with one mL of the homogenized suspension containing approximately 90–150 CFU/mL and four mL of horse blood (TCS Biosciences Ltd), for a final bacterial concentration of approximately 100 CFU/bottle. BCBs were incubated in the BacT/Alert® Virtuo Microbial Detection System until signaling for positivity, or for a maximum of 12 days. Time to positivity was recorded. When growth was detected, one drop (≈50 μl) of the BCB medium was subcultured on suitable media to confirm pure culture. Spiking experiments were performed in triplicates and two repeats were done within a time period of one month.

**Fig. 1:**
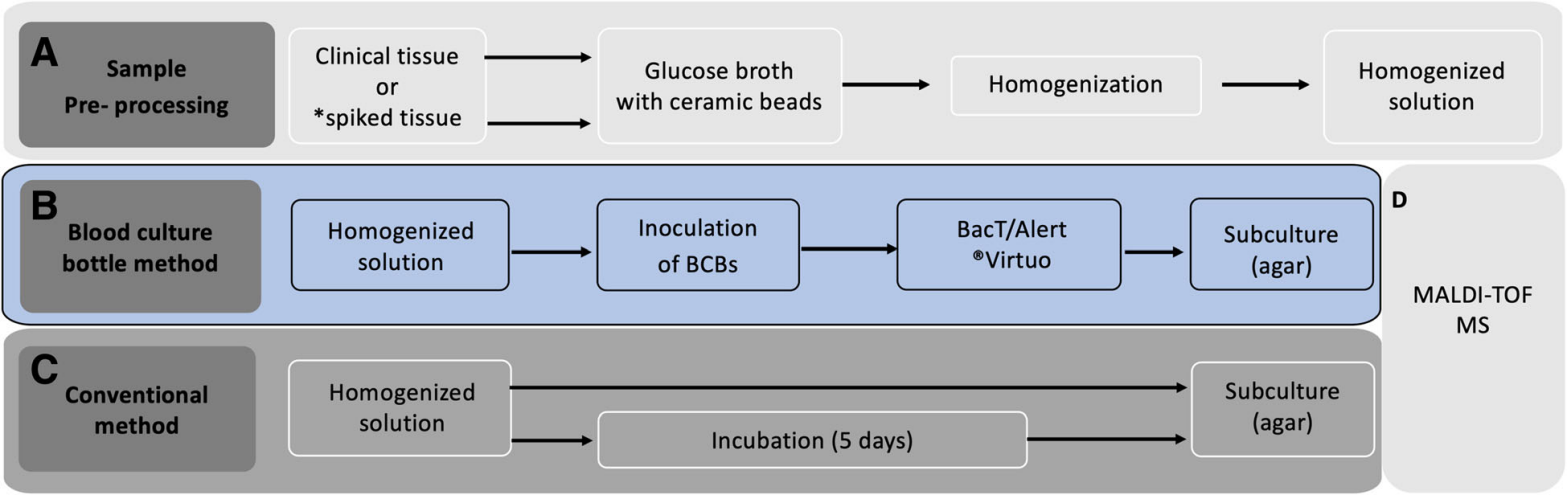
Flowchart of study design. A and D are common processes through all the methods (i.e. spiking experiments, blood culture bottle (BCB) method and conventional method). * spiked tissue was only tested using the BCB method. MALDI-TOF MS, matrix-assisted laser desorption/ionization time-of-flight mass spectrometry

In total, 36 BCBs were spiked, corresponding to 24 BacT/Alert FA plus and 12 BacT/Alert FN plus bottles. Inoculum densities, viability, and purity were checked at different time points during the process by standard microbiological methods. Tissue sterility control was performed for each bottle type by adding sterilized tissue and horse blood and incubating the bottles for 12 days as mentioned above. An overview of the process is provided in Fig. 1.

### Analysis of clinical specimens by the conventional and the BCB method

The BCB based method was run in parallel with the conventional diagnostic method. For the conventional method, several operators in the clinical microbiological laboratory were involved in reading of broth and agar plates as part of the routine procedures, while in the BCB method, only one person was involved in the experiments. For both methods, each tissue specimen (≈1 cm^3^) inoculated in four mL of glucose broth containing five ceramic beads, was homogenized in a FastPrep-24 instrument at 6.0 M/sec (meters per second) for 40 s (Fig. 1).

In the conventional method, 0.1 mL of the previously homogenized solution was inoculated onto a set of agar plates: blood, lactose, chocolate, Sabouraud and anaerobic blood agar (Fig. 1). All agar plates (aerobic and anaerobic) and the remaining homogenized solution were incubated for five days at 37 °C under aerobic conditions, with the exception of the anaerobic blood agar plates, which were incubated in an anaerobic jar system (Anoxomat® Mark II). Aerobic agar plates and the remaining broth were visually inspected daily, while the anaerobic plates where inspected after five days of incubation. The broth was inspected for opacity (almost always cloudy) and subcultured on aerobic agar for three days at 37 °C. Time to detection (TTD) for the conventional method was recorded (defined as the time until growth is detected on the subsequent agar plate cultures). Conventional microbiological methods for identification of bacteria and fungi were performed on positive subcultures.

In the BCB method, a blood culture set, corresponding to an aerobic bottle (BacT/Alert FA Plus) and an anaerobic bottle (BacT/Alert FN Plus) was inoculated with one mL of the homogenized tissue specimen using a sterile syringe. Inoculated BCBs were enriched with four mL of horse blood and incubated in the Virtuo system up to 12 days. TTD, defined as the time when the BacT/Alert® Virtuo blood culture system signaled positive, was recorded, and one drop from the bottle was subcultured on the agar plate set mentioned above.

For both methods, the identification of bacteria was done using matrix-assisted laser desorption ionization-time of flight mass spectrometry (MALDI-TOF® MS Bruker Daltonics - microflex™) and standard microbiological procedures (Fig. 1).

### Statistical analysis

Descriptive statistics for categorical variables were based on percentages and frequencies, while continuous variables were based on means, standard deviations (SDs), medians and interquartile ranges (IQRs). In addition, the McNemar’s test was used to evaluate if the differences between the methods were statistically significant. Data were analyzed utilizing GraphPad software 7.0e (GraphPad Software Inc., CA, US).

## Results

### BacT/Alert BCB system for detecting PJI pathogens - spiking

The detection rate in spiked blood culture bottles was 100% (*n* = 36) for both blood culture bottle types. BacT/Alert FA Plus bottles inoculated with aerobic bacteria flagged positive for growth before 15 h (h). The mean time to detection was 10.1 ± 2.2 h with a minimum of 7.9 h and a maximum of 14.1 h. For the BacT/Alert FN Plus bottles inoculated with anaerobic bacteria, there was a remarkable difference in TTD between the two strains tested*. C. acnes* was detected approximately nine hours later than *B. fragilis*. The mean TTD for bottles inoculated with tissue spiked with *B. fragilis* was 25.2 ± 1.1 h with a minimum TTD of 24.4 h and a maximum of 26.7 h, while for *C. acnes*, the mean TTD was 209.2 ± 18.1 h with a minimum and maximum TTD of 175.2 h and 223.2 h, respectively. No difference was observed between the two repeats performed (SD 2.3–2.2), suggesting that the method is reproducible (Additional file 1).

### BacT/Alert BCB system effectively detected pathogens from PJT specimens

After having confirmed that the BCB system can effectively detect bacteria commonly found in PJI, we next wanted to test the method on clinical specimens. Each clinical specimen (*n* = 158) was inoculated into two bottles. Therefore, 316 BCBs were included in total for the study, comprising 158 bottles per blood culture type.

Eighty (25.3%) BCBs signaled positive for growth, comprising 44 aerobic and 36 anaerobic bottles. Positive BCBs belonged to 46 (29.1%) clinical tissue specimens from 24 patients (38.70%). Two subcultures of positive BCBs were negative after culturing on agar plates and therefore classified as false positives. These were included as negative samples during all analyses.

Organisms were identified from both bottles in 73.9% of the cases, from the aerobic bottle only in 21.8%, and from the anaerobic bottle only in 4.3%. For aerobic cultures, 89% of the microorganisms were detected within 24 h and 100% within 40 h. For the anaerobic cultures, 97% of the microorganisms were detected within 24 h, and 100% within 31 h.

The mean TTD for aerobic bottles was 13.9 ± 7.8 h within a range of 3.8–39.3 h. For the anaerobic bottles, the mean, minimum and maximum TTD was 11.3 ± 5.4 h, 4 h, and 30.9 h, respectively (Additional file 1).

### BCB method yielded faster results compared with conventional method

Use of BCB for analyzing clinical tissue samples from patients with suspicion of PJI was compared with the conventional method according to method sensitivity, TTD and the bacterium (monomicrobial) or bacteria (polymicrobial) identified. For these analyses, a sample was considered positive by the BCB method when one or two of the bottle types flagged positive.

In total, 158 periprosthetic tissue samples from 62 patients were analyzed (Table 1). By using BCBs, 112 samples were negative and 46 positive, belonging to 24 patients. By the conventional method, 114 samples were negative and 44 positive corresponding to 23 patients (Table 1).

**Table 1:** Results from culturing periprosthetic tissue samples using the blood culture bottle (BCB) method and the conventional method

Tissue samples	BCB method		Conventional method	
	No. of samples (%)	No. of patients (%)	No. of samples (%)	No. of patients (%)
Positive	46 (29.1)	24 (38.7)	44 (27.8)	23 (37.1)
Negative	112 (70.9)	38 (61.3)	114 (72.2)	39 (62.9)
Total	158 (100)	62 (100)	158 (100)	62 (100)

Sensitivity was calculated without considering patient clinical data (Additional file 2). The BCB method appeared slightly more sensitive (79%) than the conventional method (76%). A two-tailed *P* value of 0.844 was obtained, which means that this difference is not statistically significant. There was an 84% agreement rate between the two methods (Table 2).

**Table 2:** Concordance between culturing clinical tissue samples using the blood culture bottle (BCB) method and the conventional method

Method		No. of tissue samples (%)
Conventional	BCB	
+	+	32 (20.2)
–	–	100 (63.3)
+	–	12 (7.6)
–	+	14 (8.9)
Total		158 (100)

**Fig. 2:**
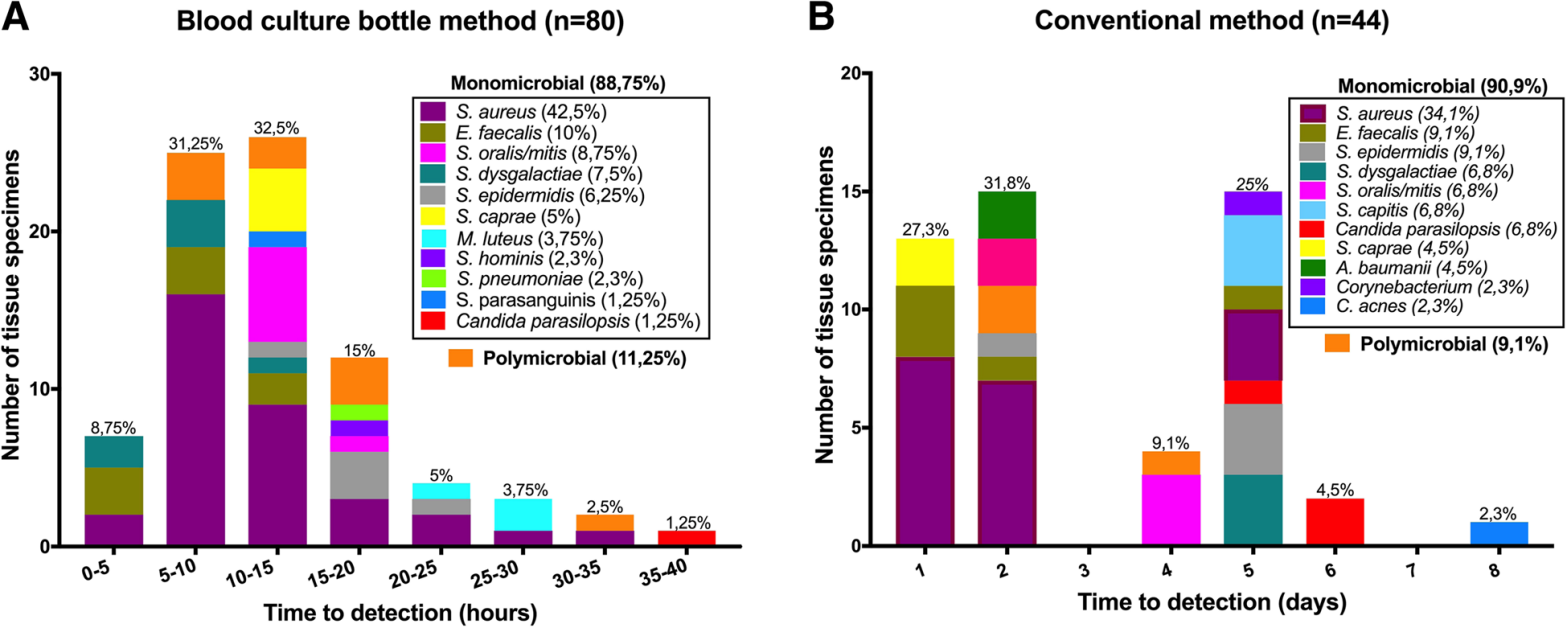
Time to detection (TTD) and organisms identified by (**a**) blood culture bottle method and (**b**) conventional method

TTD was recorded for the conventional method through daily visual inspection of agar plates and the glucose broth for bacterial growth. Fifty-nine percent of the bacteria were detected within the first 48 h, consisting of 27 and 32% of bacteria detected on the first and second day of incubation, respectively. The TTD was significantly reduced using the BacT/Alert FA plus and BacT/Alert FN Plus bottles compared to the conventional method. More than 80% of the bacteria were detected in less than 20 h using BCB, compared to the 5 days needed to obtain a similar percentage by the conventional method (Fig. 2).

### Microbiology

In total, 46 out of 158 prosthetic joint tissue (PJT) specimens belonging to 24/62 (38.7%) patients had microorganisms detected by BCBs (Table 1). Positive samples were mostly monomicrobial (88.7%), with a low rate of polymicrobial (11.3%). The two methods gave similar bacterial species in 89% of the positive samples, while 11% of the samples gave different bacterial species. The most prevalent microorganisms found using both methods, were *Staphylococcus aureus*, *Enterococcus faecalis*, *Streptococcus mitis/oralis*, *Streptococcus dysgalactiae* and *Staphylococcus epidermidis* (Fig. 2, Additional file 3).

## Discussion

Conventional microbiological culture remains the benchmark in PJI diagnosis, despite the longer time period associated with culture-based methods. Alternative approaches that are both effective and practical should be considered for use in the clinical routine setting [25].

Here, we present an evaluation of the use of the BacT/Alert® Virtuo blood culture system for microbiological analyses of periprosthetic tissue specimens as a tool that can be beneficially used for improving and accelerating the diagnosis of PJI. Our analyses included spiked and clinical samples, as well as a comparison of the BCB method with the local routine diagnostic method, for TTD and organism(s) identification. The translation of this test system into clinical application at the local level was the main goal of the project.

Currently, a microbiological definition of PJI is the isolation of two or more identical isolates from multiple specimens, or the isolation of one highly virulent organism from a single sample [9]. During this study, we worked with excess material from the clinical microbiology laboratory and the number of specimens accessible per patient varied from 1 to 5 (mean of 2.5). Thus, we have sampled a sub-optimal number of tissue specimens which may have led to diminished sensitivity. According to Peel et al. [26] three PJT specimens obtained and inoculated into BCBs will give the greatest accuracy of PJI diagnosis. Alternatively, four PJT specimens should be obtained and cultured using standard plate and broth cultures. We only evaluated the microbiological status of each sample regardless of the clinical and histopathological requirements needed to be catalogued as a true PJI.

In microbiological diagnostics, culture media and incubation time might have a high influence on test sensitivity. BCBs from different manufacturers have different compositions which could influence their performance [27]. Most of the studies using BCBs for diagnosis of PJI have used the BD BACTEC BCB system [2, 16, 22, 25, 28]. For the BacT/Alert system, only evaluation of synovial fluid has been reported [17, 28]. To the best of our knowledge, this is the first study evaluating the BacT/Alert system for culturing PJT specimens. This is relevant since there are many routine laboratories working with the BacT/Alert system from bioMérieux that could favorably implement this method into their routine procedures.

The optimal BCB incubation time is a matter of debate [29]. Commonly, most BCBs with samples obtained from sterile sites are incubated for five days [30]. Some studies suggest prolonging the incubation time up to 14 days, to increase the method sensitivity for anaerobes and slow-growing bacteria [30, 31], while others report that longer incubation time does not increase the method sensitivity [32]. In our study, BCBs were incubated for a period of up to 12 days. During our experiments, most of the bacteria could be detected within the first two days of incubation (Fig. 2), except for the spiked C*. acnes,* which needed a mean time of 209 h (8.7 days) to be detected. In the analysis of clinical specimens, just one clinical sample contained *C. acnes* by the conventional method (possible contamination) while there was not a single sample positive for *C. acnes* or other anaerobic bacteria, using the BCB method.

Recent studies have reported that the BacT/Alert FN Plus performed poorly with regard to TTD for anaerobic bacteria including *C. acnes* [24, 33]. In our case, since the spiked anaerobic bacteria *C. acnes* (clinical strain) and *B. fragilis* (ATCC 25285) could be detected by the BacT/Alert® Virtuo system, the possible lack of sensitivity for the BCB method was discarded. Instead, lack of anaerobic bacteria in the clinical tissue samples may be due to a low bacterial load, to the absence of bacteria in the sample, or to inadequate sample storage and transport (anaerobic device was not used). Overall, our results suggest that BacT/Alert® Virtuo system is able to detect relevant bacteria for PJI, including anaerobic bacteria, and that longer incubation times beyond eight days may increase the detection rate of *C. acnes.*

Despite the results mentioned above, at the general clinical level, long incubation times are not convenient in the routine clinical setting. Early results are significant for the patient as well as for the clinician. *C. acnes* is also a common contaminant of bacterial cultures and its role in PJI is not well defined [30]. We conclude that further research is needed before implementation of long incubation times to increase the detection rate of low prevalent slow-growing bacteria such as *C. acnes*.

In this study, we also compared the BCB method and the local conventional method. Use of BCBs yielded faster results than the conventional method. Shorter time to detection, when using a BCB system, has been reported earlier for different sample types (body fluids and tissue) [20, 26, 34]. Peel et al. [12] compared a BCB system with standard agar and thioglycolate broth culture, yielding faster results using BCBs and showing a 47% improvement of sensitivity.

Additionally, we have used horse blood as enrichment supplement for the BCBs, which has previously been shown to produce high positivity rates and shortening of time to detection [35]. The use of horse blood does not significantly influence the performance of blood culture systems [33, 36].

Also, the BCB method was as specific as the use of agar and broth. These findings are very interesting for cases in which rapid diagnostic methods are applied directly from positive BCBs, shortening the time for pathogen identification and to determine antimicrobial susceptibility. The BCB method was shown to be reproducible during spiking experiments, documented by the fact that there were no differences observed in the TTD from the repeats performed at different time points (Additional file 1).

*S. aureus*, *E. faecalis* and coagulase-negative staphylococci are among the most common causes of PJI [3, 12], findings that agree with our results. Less commonly recognized pathogens, such as *S. pneumoniae* and *Candida parapsilosis* have also been confirmed to be associated with PJI [37–39].

The BCB method and the conventional method were concordant in most cases. However, in some cases different microbiological agents were identified comparing the two methods. This finding may indicate a higher sensitivity of the BCB method for detection of polymicrobial samples, since a higher number of species was found in samples sub-cultured from positive BCBs (Additional file 3). This result is similar to the report by Velay et al. [22] using the BACTEC BCB system. In cases where the methods yielded different microorganism(s), discrepancies were analyzed (when possible) by comparing the results obtained from other samples belonging to the same patient and/or by the presence of a virulent bacterium (e.g. *S. aureus*). We conclude that in most of the cases, use of the BCB method was more accurate.

There are certain disadvantages using BCBs, e.g. cost of BCBs, cost of waste disposal and capacity problems [6, 26]. There is also a contamination risk when inoculating the homogenized material and horse blood into the bottles, and careful manipulation should be applied.

Our study had some technical limitations. (1) Low total number of clinical samples collected (*n* = 158) and limited number of samples per patient (mean 2.5); (2) Limited clinical data about the patients making it difficult to define a sample selection criterion to distinguish between true PJI and contaminations; (3) the inoculum volume for the BCB (1 mL) was ten times higher than that inoculated onto the agar plates (0,1 mL) which could partially explain the faster detection rate observed with the BCB method, and (4) As for the time of detection, comparative studies of this type contain a bias related to the reading frequency which differed between the two methods. In the BCB method, reading was done automatically every 10 min, whereas in the conventional method, reading was performed manually only once daily to comply with local laboratory practice. This confirms the utility and advantages of the BCB Virtuo system, including automatization, mechanical loading and unloading of bottles, faster detection of microorganisms present in the samples, minimizing the risk of contamination, in addition to reduce labor requirements in the clinical laboratory [23].

## Conclusion

In summary, we present a laboratory procedure that may be an important tool in the diagnosis of PJI. We recommend using the BacT/ALERT® BCB system for culturing periprosthetic tissue as a laboratory procedure that can reliably and rapidly detect bacteria commonly found in PJI, thus facilitating early clinical decision making.

## Additional files

Additional file 1: **Table S1.** Analysis of Time to detection (TTD) obtained from the spiking experiments. **Figure S1.** Boxplots from analysis of Time to detection (TTD) obtained from the spiking experiments. (DOCX 354 kb)

Additional file 2: **Table S2.** Conventional and BCB method sensitivity. (DOCX 13 kb)

Additional file 3: **Table S3.** Organism(s) identified by the blood culture bottle (BCB) method and the conventional method. (DOCX 96 kb)

## Data Availability

The datasets used and/or analyzed during the current study are available from the corresponding author on reasonable request.

## Abbreviations

ATCC: American type culture collection
BCB: Blood culture bottle
CFU: Colony forming units
IQRS: Interquartile ranges
MALDI-TOF MS: Matrix-assisted laser desorption/ionization time-of-flight mass spectrometry
PJI: Prosthetic joint infection
PJT: Prosthetic joint tissue
SD: Standard deviation
TTD: Time to detection
