# Supplementary material for: Culturing periprosthetic tissue in BacT/Alert® Virtuo blood culture system leads to improved and faster detection of prosthetic joint infections
Source: BMC Infect Dis. 2019 Jul 10;19:607. doi: 10.1186/s12879-019-4206-x (PMC6621959; doi:10.1186/s12879-019-4206-x)
Supplement: Supplementary file 1 — Table S1. Analysis of Time to detection (TTD) obtained from the spiking experiments. Figure S1. Boxplots from analysis of Time to detection (TTD) obtained from the spiking experiments. (DOCX 354 kb) [file 12879_2019_4206_MOESM1_ESM.docx]

**Additional file 1: Table S1.** Analysis of Time to detection (TTD) obtained from the spiking experiments.

**Additional file 1: Figure S1.** Boxplots from the analysis of Time to detection (TTD) obtained from the spiking experiments.

**Figure S1:** Boxplots from the analysis of Time to detection (TTD) obtained from the spiking experiments. **a)** Time to detection for the BacT/Alert FA Plus bottles inoculated with the aerobic bacteria; **b)** Time to detection for the BacT/Alert FN Plus bottles inoculated with *B. fragilis*; **c)** Time to detection for the BacT/Alert FN Plus bottles inoculated with *P. acnes*.
