# Supplementary material for: Culturing periprosthetic tissue in BacT/Alert® Virtuo blood culture system leads to improved and faster detection of prosthetic joint infections
Source: BMC Infect Dis. 2019 Jul 10;19:607. doi: 10.1186/s12879-019-4206-x (PMC6621959; doi:10.1186/s12879-019-4206-x)
Supplement: Supplementary file 2 — Table S2. Conventional and BCB method sensitivity. (DOCX 13 kb) [file 12879_2019_4206_MOESM2_ESM.docx]

**Additional file 2: Table S2.** Conventional and BacT/Alert blood culture system sensitivity

| **Method** | **True positives** | **Sensitivity** |
| --- | --- | --- |
| Conventional | (32+12) / (44+14) x100 | 75,86% |
| BacT/Alert blood culture system | (32+14) / (46+12) x100 | 79,31% |
