# Supplementary material for: Culturing periprosthetic tissue in BacT/Alert® Virtuo blood culture system leads to improved and faster detection of prosthetic joint infections
Source: BMC Infect Dis. 2019 Jul 10;19:607. doi: 10.1186/s12879-019-4206-x (PMC6621959; doi:10.1186/s12879-019-4206-x)
Supplement: Supplementary file 3 — Table S3. Organism(s) identified by the blood culture bottle (BCB) method and the conventional method. (DOCX 96 kb) [file 12879_2019_4206_MOESM3_ESM.docx]

**Additional file 3: Table S3.** Organism(s) identified by the blood culture bottle (BCB) method and the conventional method.

*FA: Aerobic bottle BacT/Alert FA Plus, FN: Anaerobic bottle BacT/Alert FN Plus
